# Discrepancies in the management of acquired cold contact urticaria: Results of a French-speaking urticaria experts questionnaire survey

**DOI:** 10.1016/j.waojou.2022.100688

**Published:** 2022-08-21

**Authors:** Aurélie Du-Thanh, Angèle Soria, Emmanuelle Amsler, Antoine Badaoui, Marie-Sylvie Doutre, Germaine Gabison, Claire Bernier, Delphine Staumont-Sallé, Florence Hacard, Florence Castelain, Anne-Sophie Darrigade, Gilbert Patrice ML Tapsoba, Marie-Elodie Sarre, Pascale Mathelier-Fusade, Juliette Delaunay, Pauline Pralong, Annick Barbaud, Frédéric Dezoteux, Catherine Trémeau-Martinage, Zhary Bachtarzi, Frédéric Augey, the Urticaria Group of the French Society of Dermatology

**Affiliations:** a Dermatology, University of Montpellier, Montpellier, France; b Sorbonne Université, INSERM, Institut Pierre Louis d’Epidémiologie et de Santé, Publique, AP-HP Sorbonne Université, Hôpital Tenon, Département de dermatologie et allergologie, F75020, Paris, France; c Cabinet de Dermatologie et Allergologie, 14 rue de Chateaudun, 75009 Paris, France; d Dermatology, University Hospital of Bordeaux, Bordeaux, France; e Dermatology, APHP, Paris, France; f Dermatology, University Hospital of Nantes, Nantes, France; g Dermatology, University Hospital of Lille, Lille, France; h Dermatology, University Hospital of Lyon-Sud, Pierre-Benite, France; i Dermatology, University Hospital of Besançon, Besançon, France; j Dermatology, University Hospital, Yalgado Ouedraogo, Ouagadougou, Burkina Faso; k Dermatology, University Hospital of Angers, Angers, France; l Dermatology, University Hospital of Grenoble, Grenoble, France; m Cabinet de Dermatologie, 4 rue de la Placette, Portet sur Garonne, France; n Cabinet d’Allergologie, Cité du 5 juillet 1962, Constantine, Algeria

**Keywords:** Acquired cold contact urticaria, Recommendations

## Abstract

Acquired cold contact urticaria (ACU) is a putatively serious condition, because of the risk of anaphylactic shock whenever patients are massively exposed to cold atmosphere/water, raising the question of the prescription of an “emergency kit” with oral antihistamines and epinephrine auto-injector. We performed an online survey to evaluate how French-speaking urticaria experts manage ACU. According to the 2016 consensus recommendations on chronic inducible urticarias, all the participants perform at least 1 of the available provocation tests and 84.2%, 77.8%, and 88.9% prescribe on-label use of second generation anti-H1 antihistamines (2GAH1) as a first line treatment, updosed 2GAH1 as a second line treatment, and omalizumab as a third line treatment, respectively. Interestingly, 44.4% of the practitioners always prescribe a continuous background treatment, versus 11.1% prescribing only on-demand therapy. Also, 11.7% of participants always prescribe an epinephrine auto-injector, 70.6% sometimes do, and 17.6% never do. Finally, 89.5% authorize swimming under strict conditions but 36.8% and 68.4% contra-indicate other water sports and occupational cold exposure, respectively.

## Introduction

Acquired cold contact urticaria (ACU) is the second most common subset of chronic inducible urticaria (CIndU), with an estimated annual incidence of 0.05%, a predominance in women, a long duration (4.8–7.9 years), and its management is based on second generation anti-H1 antihistamines (2GA-H1).^1^, ^2^, ^3^ When ACU is refractory to updosed 2GAH1, omalizumab, a monoclonal anti-IgE antibody indicated for the treatment of chronic spontaneous urticaria, is proposed in the 2016 consensus re-commendations for CIndU^2^ and sometimes successfully used off-label.^4^ Hopefully, therapeutic trials are currently assessing the benefit and tolerance of several new drugs.

Management of ACU may differ according to its severity. Wanderer et al^5^ classified ACU type I for localized urticaria and/or angioedema, type II for generalized urticaria and/or angioedema without hypotensive or respiratory symptoms, and type III for severe systemic reactions with ≥1 episode suggesting respiratory distress (wheezing or short-ness of breath) or hypotension (dizziness, sensation of fainting, disorientation, or shock). Type III ACU was reported in 18.6% of 415 pediatric patients.^6^ Accordingly, Wanderer et al recommend to prescribe an epinephrine auto-injector in type III ACU patients. Additionally, Bizjak et al proposed new criteria for adrenaline auto-injector prescription: previous cold-induced anaphylaxis, or no previous cold-induced anaphylaxis but the patient reports cold-induced angioedema, oropharyngeal/laryngeal symptoms, itchy earlobes.^7^

In order to evaluate the specificities in diagnosis and therapeutic management of ACU by French-speaking experts, we performed a questionnaire survey with contributors of the Urticaria Group of the French Society of Dermatology (GUS).

## Methods

The questionnaire survey was available online between October and December 2020 on the Sondageonline website for all the 43 members of the GUS. The questionnaire covered 20 items including the frequency of ACU in their daily practice, of type II or III ACU, or even any known ACU-related death in their patients, the diagnosis methods, the use and prescription of antihistamines, omalizumab, the prescription of epinephrine auto-injector, and the basic recommendations to the patients for daily life or occupational activities.

## Results and discussion

Of the GUS members, 44.2% (n = 19) completed the survey. They were representative of the French metropolitan territory with both coastal cities and cities nearby mountains, and 2 participants were from Burkina Faso and Algeria. All but 1 were dermatologists.

ACU seems an uncommon reason for consultation, even in a dedicated consultation for chronic urticaria, since despite the high specialization of participants, 61.1% of the participants see less than 10 new cases of ACU per year. Although no ACU-related death was reported, 47.4% of the participants have had patients with type III ACU.

To perform accurate ACU diagnosis, all the participants perform any of the available provocation tests (Table 1): 94.7% of the participants use the ice cube testing with a contact time of 5 minutes, but provocation times may have been shortened down to 1 minute or lengthened up to 10 minutes according to the alleged severity of ACU; whereas, 10.5% use a TempTest®, a device allowing to identify individual critical temperature thresholds. Up to 42.1% perform an immersion test (hand and/or forearm in 5–10 °C water for 10 minutes), mainly as a second line provocation test. Once ACU is confirmed, 52.6% of practitioners repeat the provocation test regularly to assess both the evolution of the disease and response to treatment and therefore adapt management (see Table 2).

**Table 1:** French-speaking experts practice regarding the 2016 consensus recommendations for ACU. 2GAH1 2nd generation anti-H1 antihistamines.

2016 consensus recommendations^2^	French-speaking experts practice
Diagnosis methods	
Ice cube testing	94.7%
Immersion test	42.1%
Temptest®	10.5%
Treatment options	
2GAH1 as 1st line	84.2%
Updosed 2GAH1 as 2nd line	77.8%
Omalizumab as 3rd line	88.9%

**Table 2:** Specificities in the management of ACU in French-speaking experts practice.

Management of ACU	Percentage of practicians
Prescription of an epinephrine auto-injector	Up to 82.3% (including 11.7% ‘always’)

Authorization of swimming activities	89.5% but 73.7% under strict restrictions^a^
Contra-indication of occupational cold exposure	68.4%
Recommendation to warm infusion fluids	42.1%
Systematic contra-indication of snow sports	0%
Systematic contra-indication of intake of cold food/drinks	50% if patient reports a history of cold anaphylaxis with cold food/drink intake only


a Including never swimming alone (84.2%), entering the water gradually (78.9%), taking prophylactic 2GAH1 (52.6%), swimming only where you can be within your depth (42.1%), and having an emergency kit (21.0%).

Regarding treatment modalities of ACU, 44.4% of the practitioners always prescribe a continuous background treatment, while 11.1% always prescribe on-demand therapy only, and 44.4% adapt their prescription to the medical history of each patient. According to the evidence for treatment options for CIndUs described by Magerl et al,^2^ 84.2% of the participants prescribe on-label use of 2GAH1 as a first line treatment, 77.8% increase the dosage of 2GAH1 as a second line treatment, and 88.9% prescribe off-label omalizumab as a third-line treatment. Only 11.1% add montelukast to on-label use of 2GAH1.

Regarding the specificities in the management of ACU (see Table 2), 17.6% never prescribe an epinephrine auto-injector, 11.7% of the participants always do, and 70.6% sometimes do. In the latter, this prescription is conditioned by a history of a previous cold anaphylaxis in 94.1% of the participants, by the presence of angioedema with cold food in 82.3%, and in case of repeated occupational cold exposure or for sports in 70.6% and 58.8% of the participants, respectively. However, this prescription did not differ in practitioners from cities in Northern France versus Southern France and Africa or practicing in coastal versus non-coastal cities (supplementary Table 1).

Participants gave highly variable advice to their ACU patients regarding risky situations. Indeed, according to the literature, swimming is considered the most common risky situation, as well as water sports. However, only 10.5% of practitioners systematically contraindicate swimming, while 73.7% authorize it providing strict conditions, such as never swimming alone (84.2%), entering the water gradually (78.9%), taking prophylactic 2GAH1 (52.6%), swimming only where you can be within your depth (42.1%), and having an epinephrine auto-injector (21.0%). To note, 15.8% of practitioners do not prescribe any prophylactic antihistamines before swimming activities, either because they are not considered useful or because they could mask hives as a warning symptom of anaphylaxis.

If a patient reports a previous episode of angioedema with cold food intake, half of the practitioners systematically contraindicate further intake of cold food or drinks and 82.3% prescribe an epinephrine auto-injector. The other half of the practitioners authorize cold food but only with prophylactic 2GAH1.

Regarding specific recommendations about infusions of cold fluids, 42.1% of practitioners always recommend their warming.

Regarding other risky situations, 68.4% of practitioners systematically contraindicate occupational exposure to cold atmosphere but none systematically contraindicates snow sports or mountain hikes. Thus, snow sports seem less risky situations than swimming activities for French-speaking experts. Accordingly, in Southern America, a significant difference has been reported according to the altitude and location regarding ACU prevalence,^8^ which was 3.3 times higher in a city with a temperate/warm climate by the seaside versus a city located more than 2600 m above sea level (p = 0.02).

## Conclusion

Despite discrepancies in the specific recommendations regarding ACU management, up to 82.3% of the participants would prescribe an epinephrine auto-injector, and 89.5% do not systematically contra-indicate swimming activities providing some strict restrictions. These preliminary results justify the need for specific studies aimed at developing practical recommendations for the management of ACU, beside the development of new therapeutic options.

## Funding

This research did not receive any specific grant from funding agencies in the public, commercial, or not-for-profit sectors.

## Availability of data and materials

The submitted questionnaire is available as a supplementary material.

## Authors’ contributions

• Substantial contributions to the conception or design of the work; or the acquisition, analysis, or interpretation of data for the work: all the authors.

• Drafting the work or revising it critically for important intellectual content: all the authors.

• Final approval of the version to be published: all the authors.

• Agreement to be accountable for all aspects of the work in ensuring that questions related to the accuracy or integrity of any part of the work are appropriately investigated and resolved: all the authors.

## Authors’ consent for publication

All authors approve the manuscript and consent for publication.

## Ethics approval

Not applicable.

## Declaration of competing interest

ADT: speaker, consultant, grants, accomodations fees, investigator (10.13039/100004336Novartis SAS, Lilly, Janssen, 10.13039/100006483Abbvie, 10.13039/100004319Pfizer, LeoPharma, 10.13039/100004339Sanofi, Biocryst, Takeda);

AS: consultant for Novartis SAS, Abbvie, Sanofi, Lilly, LeoPharma, Pfizer.

EA: employee of Product life group, consultant and speaker for Novartis, speaker for Sanofi.

MSD: investigator for Novartis.

GG: none to declare.

CB: speaker for 10.13039/100004336Novartis, accommodations fees from Novartis.

DSS: investigator, consultant and speaker for Novartis.

FH: speaker, consultant for Novartis.

FC: none to declare.

ASD: speaker for 10.13039/100004336Novartis, accommodations fees from Novartis.

GP ML T: none to declare.

MES: none to declare.

PMF: none to declare.

JD: none to declare.

PP: none to declare.

AB: speaker, principal investigator for Novartis.

FD: none to declare.

CTM: accommodations fees from 10.13039/100004336Novartis, speaker for Novartis.

ZB: none to declare.

FA: accommodations fees from Novartis.
